# The impact of acid mine drainage on nitrogen-fixing microorganisms in rice root zone soil

**DOI:** 10.71150/jm.2505004

**Published:** 2026-01-31

**Authors:** Shengni Tian, Penghui Zhang, Qin Zhang, Yupeng Chen, Caijuan Sun, Dan Huang, Wenye Zhang, Mingzhu Zhang

**Affiliations:** School of Life Sciences, Anhui Agricultural University, Hefei 230036, P. R. China

**Keywords:** acid mine drainage, rice root zone soil, nitrogen-fixing microorganisms, heavy metals

## Abstract

Acid mine drainage (AMD) poses a serious threat to rice paddy ecosystems, yet its impact on the composition and dynamics of soil nitrogen-fixing microorganisms remains poorly understood. In this study, a pot experiment was conducted using paddy soil collected from a mining area under three pollution treatments, to analyze changes in the structure of the nitrogen-fixing microbial community across different growth stages and treatments. The results showed that AMD irrigation led to soil acidification, sulfate accumulation, and a significant reduction in the diversity of nitrogen-fixing microorganisms in the root zone. Compared to the control, the Shannon index decreased by 11.65–24.79% in contaminated soil. LEfSe analysis indicated that AMD enriched metal-tolerant and sulfate-resistant microbial taxa. Irrigation with clean water was insufficient to fully restore the soil environment. The assembly process of the AMD soil community was governed solely by stochastic processes, indicating structural instability of the community. This study suggests that remediation strategies should prioritize neutralizing acidity and restoring nutrient balance to support the stability and recovery of nitrogen-fixing microorganisms. These findings provide new insight into how AMD disrupts diazotrophic community assembly, with direct implications for paddy soil restoration.

## Introduction

AMD is a severely polluting wastewater characterized by high acidity, sulfate, and heavy metals. While mining supports economic development, AMD contamination degrades soil quality and disrupts the functions of microorganisms (Dou et al., 2023; Jiang et al., 2022; Xu et al., 2020). Its hydrological migration destabilizes aquatic ecosystems and jeopardizes water quality for human consumption and irrigation (Masindi et al., 2022). Microbial oxidation of exposed slag forms AMD under the weathering effect of air and water (Zhang et al., 2023). Globally, long-abandoned mining sites continue to expose surrounding environments to AMD contamination (Graham and Knelman, 2023; Xin et al., 2021). There are approximately 558,000 abandoned/closed mines in the United States (Moodley et al., 2018), a large number of which have produced AMD and damaged an estimated 23,000 kilometers of streams and rivers (Park et al., 2019). It is reported that the annual mine drainage volume of China is about 700 million tons, of which AMD accounts for more than 50% (Jiao et al., 2023). In the management of mine drainage, China requires that the recycling rate of mine wastewater must reach over 65%, and the discharge into agricultural and forestry areas must meet water quality requirements.

Nitrogen-fixing microorganisms are the core drivers of soil nitrogen cycling, contributing 40–70 Tg of reactive nitrogen annually to agricultural systems through biological nitrogen fixation mediated by the *nifH* gene (Ladha et al., 2022; Mahboob et al., 2023; Xu and Wang, 2023). Their community structure and diversity directly influence soil ecological functions, making them a potential indicator for assessing soil quality (Wolińska et al., 2016). Research demonstrates that AMD contamination significantly alters the diversity of nitrogen-fixing microorganisms by modifying multiple environmental factors, such as pH, heavy metal concentrations, and sulfate levels (Chen et al., 2021; Distaso et al., 2022; Plaza-Cazón et al., 2021). Under heavy metal stress, tolerant Nitrogen-fixing microbial communities may develop metabolic adaptations to establish dominance (Li et al., 2023b). In addition, AMD can cause changes in soil nitrogen concentration. High concentrations of nitrogen may enhance the competitiveness of certain nitrogen-fixing microorganisms (Gao et al., 2024; Naz et al., 2022; Pan et al., 2021; Stefanowicz et al., 2020). The direct effects of AMD have been comprehensively studied. However, there remains a lack of research regarding its long-term impacts on the ecology of nitrogen-fixing microorganisms in rice.

Compared to other ecosystems, paddy soil microbial communities display unique cyclic variations driven by alternating flooded and non-flooded conditions. These wet-dry cycles not only alter nutrient composition and redox status but also facilitate the formation of distinct microbial structures under significant environmental shifts (Kimura and Asakawa, 2005). Microbial diversity and distribution of rice soils in mining areas have been emphasized in previous studies. However, biodiversity analysis may not be sufficient to capture the most important biodiversity characteristics for ecosystem functions. Studying microbial assembly processes enhances understanding of environmental impacts on nitrogen-fixing microorganisms (Dini-Andreote et al., 2015; Graham and Knelman, 2023). Microbial community formation is governed by two primary processes: deterministic and stochastic (Zhang et al., 2024). Understanding these community assembly mechanisms is essential for comprehensively elucidating how microbial communities in rice root zone soils establish and persist under AMD stress (Niu et al., 2024). Although the long-term ecological effects of AMD on nitrogen-fixing communities in rice paddies remain poorly characterized, primarily concerning microbial assembly processes, this is a gap that this study addresses.

In this study, we employed pot experiments to simulate rice root zone environments under gradient AMD contamination levels, to investigate dynamic variations in the composition, diversity, functional genes, co-occurrence networks, and assembly processes of nitrogen-fixing microorganisms within the root zone. This study provides insights into the changes in rice root-associated nitrogen-fixing bacteria under AMD-induced stress and offers implications for the restoration of contaminated paddy ecosystems.

## Materials and Methods

### Study area overview

The study area is situated within a mining zone of Zhongming Town, Tongling City, Anhui Province, China. It is characterized by a subtropical climate, featuring an annual average temperature of 16.2°C, a frost-free period of 237–258 days, and a mean relative humidity of 75% to 81%. The annual precipitation is 1,346 mm, predominantly concentrated between July and September. This research focuses on two adjacent paddy fields, separated by the S32 Tongxuan Expressway. The experimental soils were collected from two distinct paddy fields: one contaminated by acid mine drainage, and the other irrigated with normal water as a control. The AMD-contaminated field, irrigated long-term with AMD, is geographically positioned at 30°58'26" N, 118°04'35" E, while the control field utilizing uncontaminated water sources lies 3 km southwest of the polluted site (30°58'38" N, 118°04'15" E).

### Experimental design and sampling

For the experiment, due to the complex root system of rice, 13 kg of soil was placed into 1-meter-high plastic buckets, which are hereafter referred to as pots, were used as containers for the pot experiment to mitigate the constraints of conventional pots on root development. A resin-coated slow-release fertilizer (comprising 24% nitrogen [N], 6% phosphorus pentoxide [P_2_O_5_], and 10% potassium oxide [K_2_O]) was uniformly applied as a basal dose at 1 g/kg soil, followed by thorough mixing and flooding. Three experimental treatments were established: AMD irrigation of polluted soil (Treatment A), Clean water irrigation of polluted soil (Treatment B), and clean water irrigation of unpolluted soil (Treatment CK). Each treatment was replicated three times to ensure data reliability. Rice was cultivated in pots with three plant clusters per pot (two seedlings per cluster), and all pots were housed in a ventilated, rain-sheltered greenhouse to standardize environmental conditions. To maintain flooded soil conditions, daily irrigation preserved a 3–4 cm water layer. During the drainage phase, intermittent irrigation was applied after soil drying until harvest. Root zone soil samples were collected at four growth stages: seedling, tillering, heading, and maturity. Fresh soil samples were stored at -20°C for microbial DNA extraction and subsequent high-throughput sequencing, while air-dried samples were analyzed for basic physicochemical properties and heavy metal concentrations.

### Soil physicochemical property analyses

The air-dried soil samples were placed in a cool, ventilated area, then ground to remove impurities such as stones and roots. A portion of the dried soil was sieved through a 10-mesh nylon sieve for analyzing pH, NH_4_^+^-N, and NO_3_^⁻^-N, while the remaining soil was sieved through a 100-mesh nylon sieve to determine SO_4_^2-^, total phosphorus (TP), organic matter (OM), and heavy metal concentrations. Soil pH is measured using a pH meter (PHS-3CU, InsMark, China) at a soil-to-water ratio of 2.5:1. Total nitrogen (TN) is determined via the sulfuric acid digestion-Kjeldahl method. NO_3_^⁻^-N and NH_4_^+^-N are quantified using a UV spectrophotometer (T6S, Beijing Puxi GENERAL Instrument Co., Ltd, China). SO_4_^2-^ is analyzed by ion chromatography (Yang et al., 2016), organic matter (OM) by the external potassium dichromate heating method (Mabagala and Mng'ong'o, 2022), and total phosphorus (TP) via perchloric acid-sulfuric acid digestion followed by molybdenum-antimony colorimetry. For heavy metal analysis (Cu, Cd, Mn, Pb, and Zn), soil samples are digested with a mixture of HF, HClO_4_, and HNO_3_. Concentrations of Cu, Cd, Mn, Pb, and Zn in the digested solutions are quantified using an atomic absorption spectrophotometer (TAS-990, Beijing Puxi GENERAL Instrument Co., Ltd., China). Soil heavy metal pollution levels are assessed via the Nemerow pollution index (Wang et al., 2011). The index evaluates pollution by ratioing measured contaminant concentrations to regulatory standards, identifying primary pollutants and overall contamination.

### DNA extraction, *nifH* gene sequencing

Total DNA was extracted from soil samples using CTAB method (Tanase et al., 2015), and the obtained DNA was stored at -20°C. A 0.5 g aliquot of fresh soil was mixed with DNA extraction buffer (0.5 mol/L phosphate buffer, 0.5 mol/L EDTA·Na_2_, 1 mol/L Tris-HCl, 3 mol/L NaCl, 1% CTAB, pH 8.0), followed by vortex mixing and lysozyme treatment in a water bath. The mixture was subjected to multiple cycles of freezing in liquid nitrogen and heating in a boiling water bath. Subsequently, SDS and proteinase K were added for digestion, followed by centrifugation. The supernatant was then treated stepwise: incubation with pre-cooled 8 mol/L potassium acetate on ice, followed by centrifugation; extraction with phenol:chloroform:isoamyl alcohol, followed by centrifugation; extraction with chloroform:isoamyl alcohol, followed by centrifugation. The resulting aqueous phase was combined with an equal volume of PEG-NaCl, mixed thoroughly, and allowed to stand for 2 h before centrifugation to discard the supernatant. The precipitate was washed with ethanol, air-dried, and finally dissolved in TE buffer.

Using the extracted soil genomic DNA as a template, *nifH* gene fragment was amplified with primers *nifH*-F (5'-AAAGGYGGWATCGGYAARTCCACCAC-3') and *nifH*-R (5'-TTGTTSGCSGCRTACATSGCCATCAT-3') (Rösch and Bothe, 2005). The resulting amplicons were sequenced on an Illumina MiSeq PE 300 platform (Shanghai Majorbio Bio-pharm Technology Co., Ltd., China) to obtain taxonomic information of nitrogen-fixing microorganisms.

The high-quality assembled sequences from all samples were clustered into operational taxonomic units (OTUs) using the USEARCH software, with a default identity threshold of 97%. The high-quality sequences were clustered into operational taxonomic units (OTUs) at 97% identity using the USEARCH software.

### Fluorescence quantification

The *nifH* gene was amplified from soil DNA using above primers, followed by cloning into the pEASY-T3 vector and transformation into Trans1-T1 *E. coli* competent cells. Positive clones were selected via blue-white screening, cultured, and Sanger-sequenced (BGI Genomics Co., Ltd., China) to verify insert identity. Validated clones were used as templates for M13 primer-mediated re-amplification. The resulting *nifH* fragments were purified and stored at -20°C as quantitative standards for qPCR.

Quantitative real-time PCR was performed on a StepOne Real-Time PCR System (Applied Biosystems, USA). Purified plasmid DNA carrying the target gene from randomly selected clones was used as the standard. A standard curve was generated using ten-fold serial dilutions (in triplicate) of the standard plasmid with known copy numbers. Each run was repeated three times independently. The R² value for each standard curve exceeded 0.99 in all replicates. The expression level of the target gene in unknown samples was calculated based on the standard curve.

### Statistical analysis

Statistical analysis was performed using SPSS 20.0 to examine significant differences in dominant microbial taxa, the ɑ-diversity (Chao1 and Shannon indices) of nitrogen-fixing bacteria, and environmental factors using ANOVA. Line charts and box plots were created utilizing Origin 2022. Differences in nitrogen-fixing microbial richness across treatment groups were assessed via LEfSe (Linear Discriminant Analysis Effect Size), considering only values with linear discriminant analysis (LDA) scores above 3.0. The contribution of environmental factors to nitrogen-fixing microbial communities was quantified using Variance Partitioning Analysis (VPA). Additionally, a partial mantel test was conducted with the "vegan" package in R. Dominant nitrogen-fixing genera were identified, and their correlations with soil environmental factors were calculated using Spearman’s rank correlation coefficients, with relationships visualized in heatmaps. For identified nitrogen-fixing genera across samples, pairwise Spearman correlations were computed (|r| = 1, *p* < 0.05), and genus-level interaction networks were constructed. The community assembly processes were analyzed using a between-community null modeling approach by calculating the beta-nearest-taxon index (βNTI) and the Bray-Curtis-based Raup-Crick (RC_bray_) (Wang et al., 2024a). The βNTI metric is used to determine whether shifts in community composition are driven by deterministic or stochastic processes. The RCBray index is applied to further discern the specific components of stochastic assembly. NTI was calculated using the 'picante' package in R, while phylocom 4.2 was employed for βNTI analysis.

## Results

### Soil physicochemical properties

**Fig. 1.**
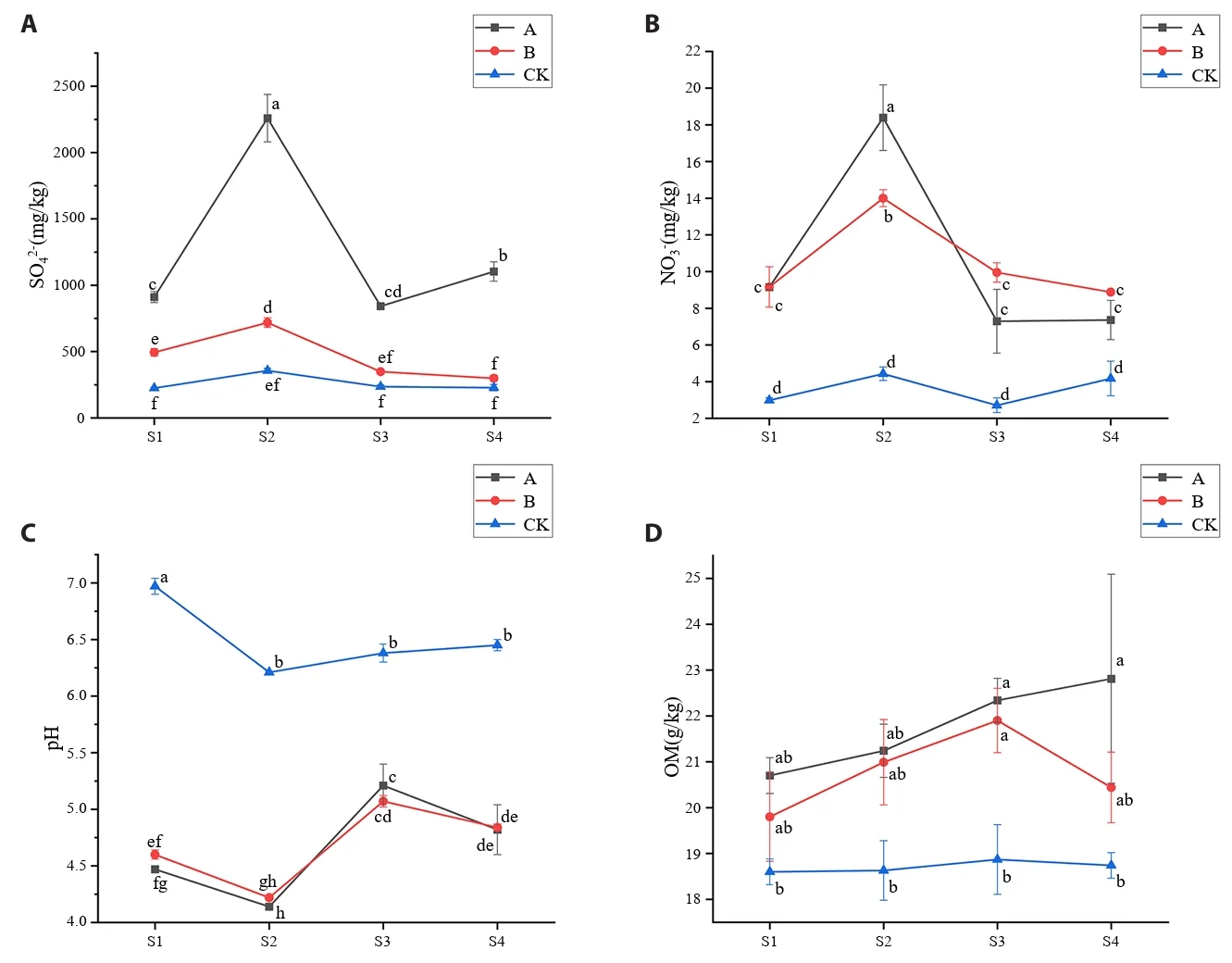
Changes in physicochemical properties of rice root zone soil at different growth stages. Different Letters above error bar indicate significant differences between samples at *p* < 0.05 using ANOVA test. (A) SO₄²⁻ content; (B) NO₃⁻ content; (C) pH; (D) Organic matter. S1: Seedling stage; S2: Tillering stage; S3: Heading stage; S4: Maturity stage.

AMD irrigation resulted in soil acidification in the root zone of rice, and the pH value was significantly (*p* < 0.05) lower than that of control soil, especially at tillering stage and ripening stage (Fig. 1A). At the tillering stage, soil contaminated with AMD exhibited significantly (*p* < 0.05) higher NO_3_^−^-N content compared to control soil (Fig. 1B). NH_4_^+^-N concentrations displayed uniform temporal patterns across all treatments, with peak values consistently observed during the tillering stage (Table S1). AMD irrigation increased the SO_4_^2-^ content in soil, especially at tillering stage, and the SO_4_^2-^ content in A treatment (AMD irrigation) was 6.33 times that of control soil (Fig. 1C). OM content in AMD contaminated soil showed an increasing trend with rice growth, but after the restoration of clean water irrigation, OM content in contaminated soil decreased (Fig. 1D). The contents of TP and TN in the soil treated by AMD increased, and the contents of TP and TN in the soil treated by A and B were higher than those in the control soil at all periods (Table S1).

The contents of Cu, Cd, Pb, and Zn in treatment A and treatment B were significantly (*p* < 0.05) higher than those in control group at different time points (Table S1). In particular, Cu content in A treatment was much higher than that in CK treatment, while Mn content in AMD-contaminated soil was significantly (*p* < 0.05) lower than that in control soil (Table S1). Compared with treatment A, the contents of Cu and Cd in soil after treatment B decreased, while the contents of Mn, Pb, and Zn in soil after treatment B had no significant (*p* > 0.05) difference with treatment A (Table S1). The Nemerow index indicated that CK soils remained ‘non-polluted’ (P_i_ ≤ 1) across all growth stages, with values falling below the background level. In soils A and B, Cu, Zn, and Cd levels were classified as ‘slightly polluted’ (1 < P_i_ ≤ 2), whereas Mn was categorized as ‘non-polluted’ (P_i_ ≤ 1). However, the index further indicated Pb pollution at 3–5 times above background levels, classifying soils A and B as ‘moderately polluted’ (Tables S2 and S3).

### Variations of nitrogen-fixing microorganisms in root zone soil

**Fig. 2.**
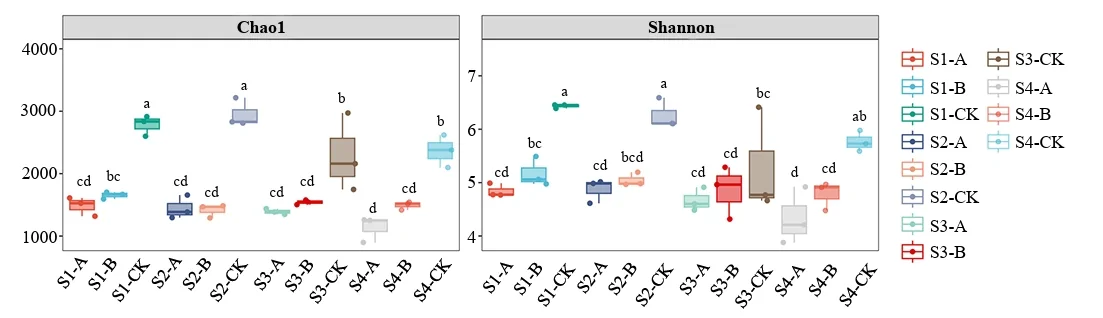
Chao1 index and Shannon index of nitrogen-fixing microorganisms in rice root zone soil samples. Different Letters above error bar indicate significant differences between samples at *p* < 0.05 using ANOVA test.

Following clustering at a 97% similarity threshold, 7,558 operational taxonomic units (OTUs) were obtained from the samples analyzed in this study (Fig. 2). The alpha-diversity of root zone nitrogen-fixing microorganisms was assessed using the Chao1 and Shannon indices. Throughout the rice growth cycle, the nitrogen-fixing microbial communities in CK soil exhibited significantly higher diversity than those in Soils A and B (*p* < 0.05). Soil B did not significantly differ from Soil A (*p* > 0.05).

According to the OTU species annotation, seven phyla of nitrogen-fixing microorganisms were identified at the phylum level. These include *Proteobacteria*, *Verrucomicrobia*, *Actinobacteria*, *Cyanobacteria*, *Firmicutes*, *Chlorobi*, and *Euryarchaeota*. *Proteobacteria* was the most abundant phylum, particularly in the control soil (CK), where its average relative abundance was the highest (exceeding 75%). In contrast, in soils A and B contaminated by acid mine wastewater, its relative abundance was above 55% and 57.83%, respectively.

**Fig. 3.**
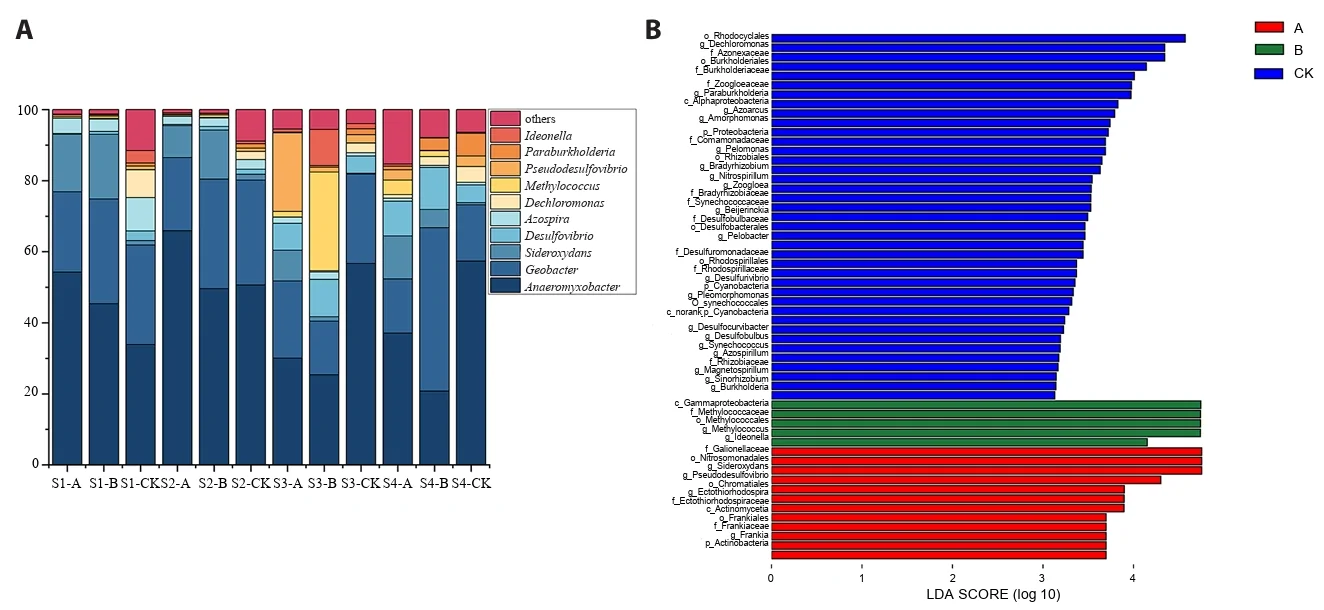
Differences in community structure and richness of nitrogen-fixing microorganisms. (A) Genus-level composition of nitrogen-fixing microorganisms across samples. (B) LEfSe was based on community difference analysis among different treatment (LDA > 3).

At the genus level, *Anaeromyxobacter*, *Geobacter*, *Sideroxydans*, *Desulfovibrio*, *Azospira*, *Dechloromonas*, *Methylococcus*, *Paraburkholderia*, *Ideonella*, and *Pseudofovibrio* were identified as dominant nitrogen-fixing bacterial genera (Fig. 3A). During the seedling and tillering stages, the average relative abundance of *Anaeromyxobacter* in soil A was higher than in soils B and CK. However, during the heading and maturity stages, *Anaeromyxobacter* abundance in CK soil surpassed that of soils A and B. The average relative abundance of *Geobacter* in soil B at the heading and maturity stages was significantly higher than in other treatments. Additionally, the relative abundance of *Dechloromonas* in CK soil was the highest and exhibited an increasing trend over time.

The variation in *nifH* gene abundance across different treatments and time points was analyzed. The results demonstrated that CK-treated soil consistently exhibited the lowest *nifH* gene abundance during each sampling period. In contrast, B-treated soil at the maturity stage showed the highest abundance, whereas A-treated soil displayed the highest values in other growth stages. Additionally, *nifH* gene abundance in B-treated soil increased progressively over time, whereas in A-treated soil, it was the lowest at the maturity stage (Table S4).

LEfSe (linear discriminant analysis effect size) was employed to assess differences in nitrogen-fixing microorganisms among treatment groups. LEfSe analysis identified 56 significantly discriminant taxa (LDA score > 3.0) across treatment groups, with Taxon-specific enrichment observed among conditions: Treatment CK exhibited the highest differentiation (39 taxa), while Treatments B and A yielded 5 and 12 unique biomarker species, respectively (Fig. 3B). At the phylum and class levels, *Cyanobacteria* (phylum) and *Alphaproteobacteria* (class) were enriched in CK soil, *Gammaproteobacteria* (class) in B soil, and *Actinobacteria* (phylum) in A soil. At the genus level, *Methylococcus* and *Ideonella* showed significant enrichment in soil B, whereas *Sideroxydans*, *Pseudodesulfovibrio*, *Ectothiorhodospira*, and *Frankia* were predominantly enriched in soil A. Additionally, *Dechloromonas*, *Paraburkholderia*, *Azoarcus*, *Amorphomonas*, *Pelomonas*, *Bradyrhizobium*, *Nitrospirillum*, and *Zoogloea* were strongly associated with CK soil (Fig. 3B).

### Effects of environmental factors on nitrogen-fixing microorganisms

**Fig. 4.**
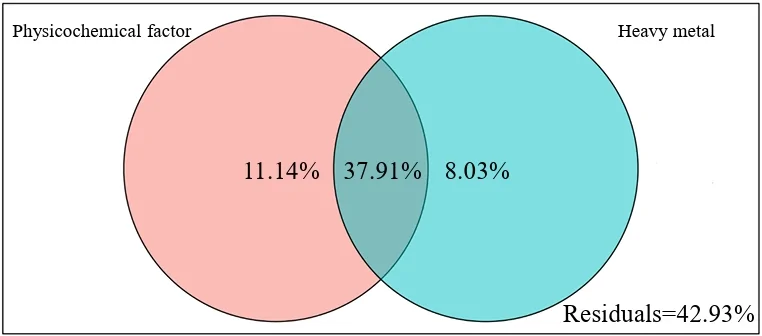
The contribution of environmental factors. Variance decomposition analysis of nitrogen-fixing microbial communities under the combined action of different environmental factors.

**Fig. 5.**
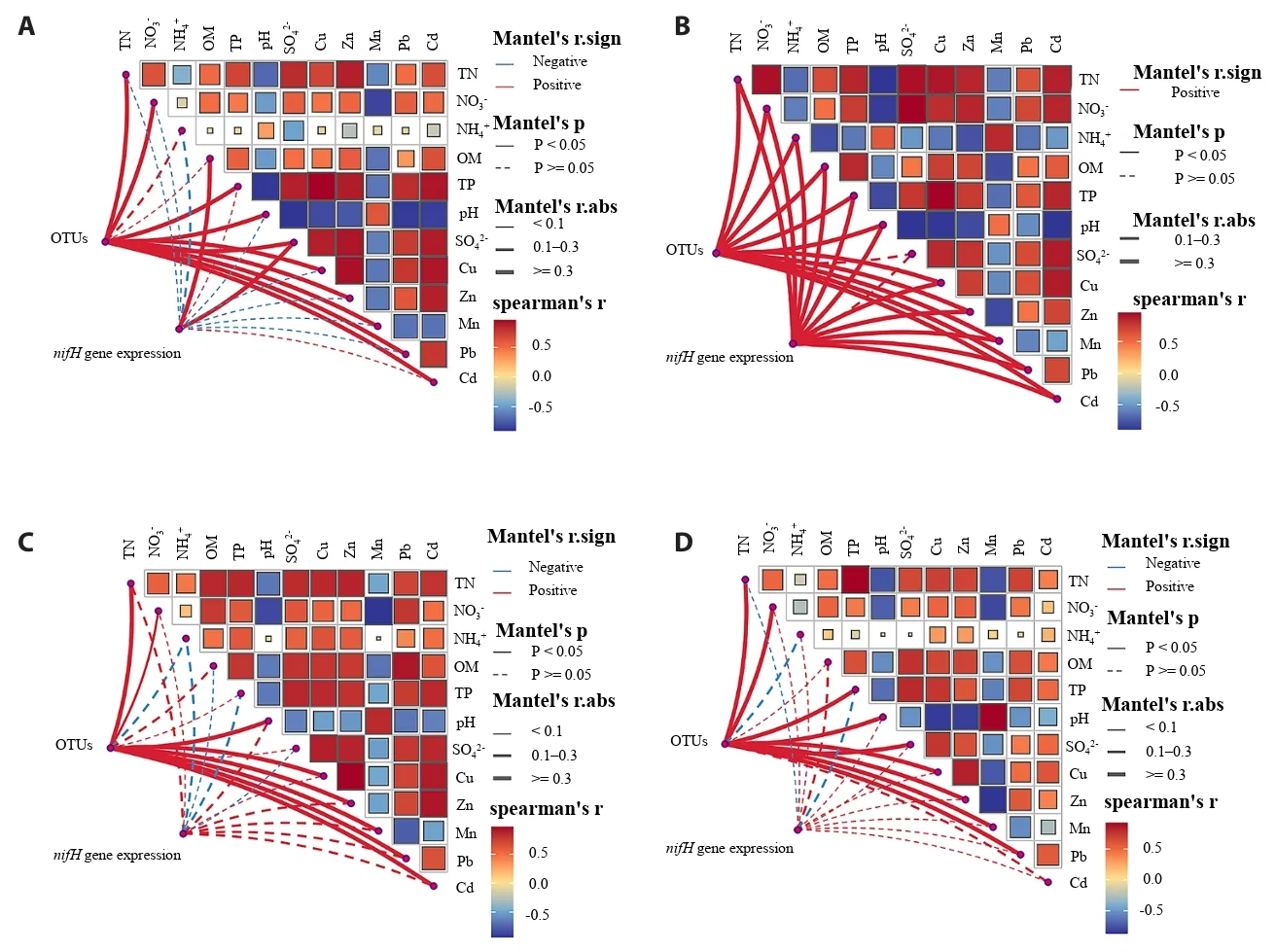
Mantel test analysis reveals correlations between OTU-level nitrogen-fixing microorganisms, *nifH* gene abundance, and rice root zone physicochemical properties. Red lines represent positive correlations, blue lines represent negative correlations, and solid lines indicate statistically significant correlations (*p* < 0.05). The width of the connecting lines corresponds to the magnitude of the correlation coefficient (r). (A) seedling stage; (B) tillering stage; (C) heading stage; (D) maturity stage.

Variance partitioning analysis (VPA) revealed that chemical properties (pH and nutrient content) independently explained 11.14% of the variance in the total bacterial community, exceeding the contribution of heavy metals (Cu, Zn, Mn, Pb, and Cd) to community variations (Fig. 4). Furthermore, the partial mantel test clarified the influence of environmental factors on nitrogen-fixing microorganisms at the OTU level and the abundance of the *nifH* gene (Fig. 5). Across all rice growth stages, nitrogen-fixing microbial communities exhibited significant (*p* < 0.05) positive correlations with soil physicochemical properties (TN, NO_3_^⁻^-N, and pH) and heavy metal concentrations (Cu, Zn, Mn, and Pb). The abundance of the *nifH* gene was significant (*p* < 0.05) positive with most chemical properties (pH, OM, TP, and TN) and the contents of heavy metals (Cu, Zn, Pb, Cd, and Mn) only at the tillering stage. At the heading and maturity stages, the abundance of the *nifH* gene exhibited no significant correlation with any of the environmental factors.

**Fig. 6.**
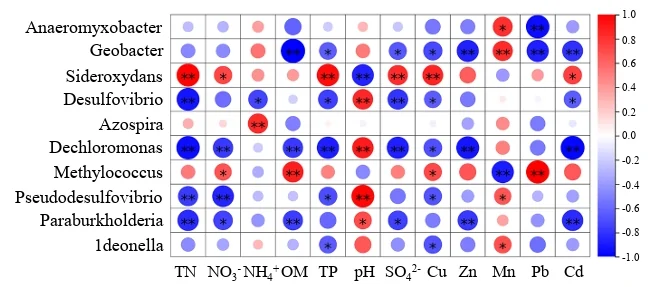
Dominant genera and environmental factor related heat map. ^*^represent *p* < 0.05; ^**^represent *p* < 0.01.

The correlation heat map was constructed to analyze the correlation between ten dominant bacteria and environmental factors. Most dominant genera showed positive correlations with pH and Mn, but negative correlations with other parameters (Fig. 6). *Sideroxydans* and *Methylococcus* were positively correlated with most environmental factors, and *Sideroxydans* was significantly (*p* < 0.05) positively correlated with TN, TP, SO_4_^2-^, NO_3_^-^-N, Cu, and Cd. *Methylococcus* was significantly (*p* < 0.05) positively correlated with NO_3_^⁻^-N, OM, Cu, and Pb. *Geobacter*, *Desulfovibrio*, *Dechloromonas*, *Pseudodesulfovibrio*, *Paraburkholderia*, and *ldeonella* are negatively correlated with most environmental factors. *Geobacter* was significantly (*p* < 0.01) negatively correlated with OM, TP, SO_4_^2-^, Cu, Zn, Pb, and Cd, *Dechloromonas* was significantly (*p* < 0.01) negatively correlated with TN, NO_3_^⁻^-N, OM, TP, SO_4_^2-^, Zn, and Cd.

### Co-occurrence and assembly of nitrogen-fixing microorganisms in root zone soil

To investigate species interactions across rice growth stages and soil treatments, co-occurrence network analysis was performed. A connection represents a strong (|r| = 1) and significant (*p* < 0.05) correlation between genera. In soils treated with A, B, and CK, nitrogen-fixing microbial communities exhibited complex interaction patterns during different rice growth stages, with positive correlations consistently outnumbering negative correlations. The nitrogen-fixing bacterial community in the CK treatment displayed the highest network complexity, characterized by the maximum number of nodes and connections, suggesting superior ecological diversity and structural stability compared to other treatments. Furthermore, across all soil treatments, the diversity of nitrogen-fixing microbial communities increased progressively as rice growth advanced, accompanied by an increase in keystone taxa and overall network complexity. However, the proportion of positive correlations among species decreased in later growth stages (Table S5).

Specifically, in the rice root zone soil treated with A (Fig. S1), *Desulfobulbus*, *Desulfurivibrio*, *Bradyrhizobium*, and *Ideonella* showed the most connection relationships in three periods, becoming the core genera of nitrogen-fixing bacteria in the A-treated soil. In the rice root zone soil treated with B (Fig. S2), *Desulfobulbus* had the most connection relationships in four periods and was the most critical nitrogen-fixing genus in the B-treated soil; *Frankia*, *Bradyrhizobium*, *Methylocystis*, and *Paraburkholderia* showed the most connection relationships in three periods. For the rice root zone soil of the CK treatment (Fig. S3), *Paraburkholderia* had the most connection relationships in four periods and became the most critical nitrogen-fixing genus in the CK-treated soil; while the five genera *Rubrivivax*, *Sideroxydans*, *Desulfovibrio*, *Azospira*, and *Sinorhizobium* also showed the most connection relationships in three periods.

**Fig. 7.**
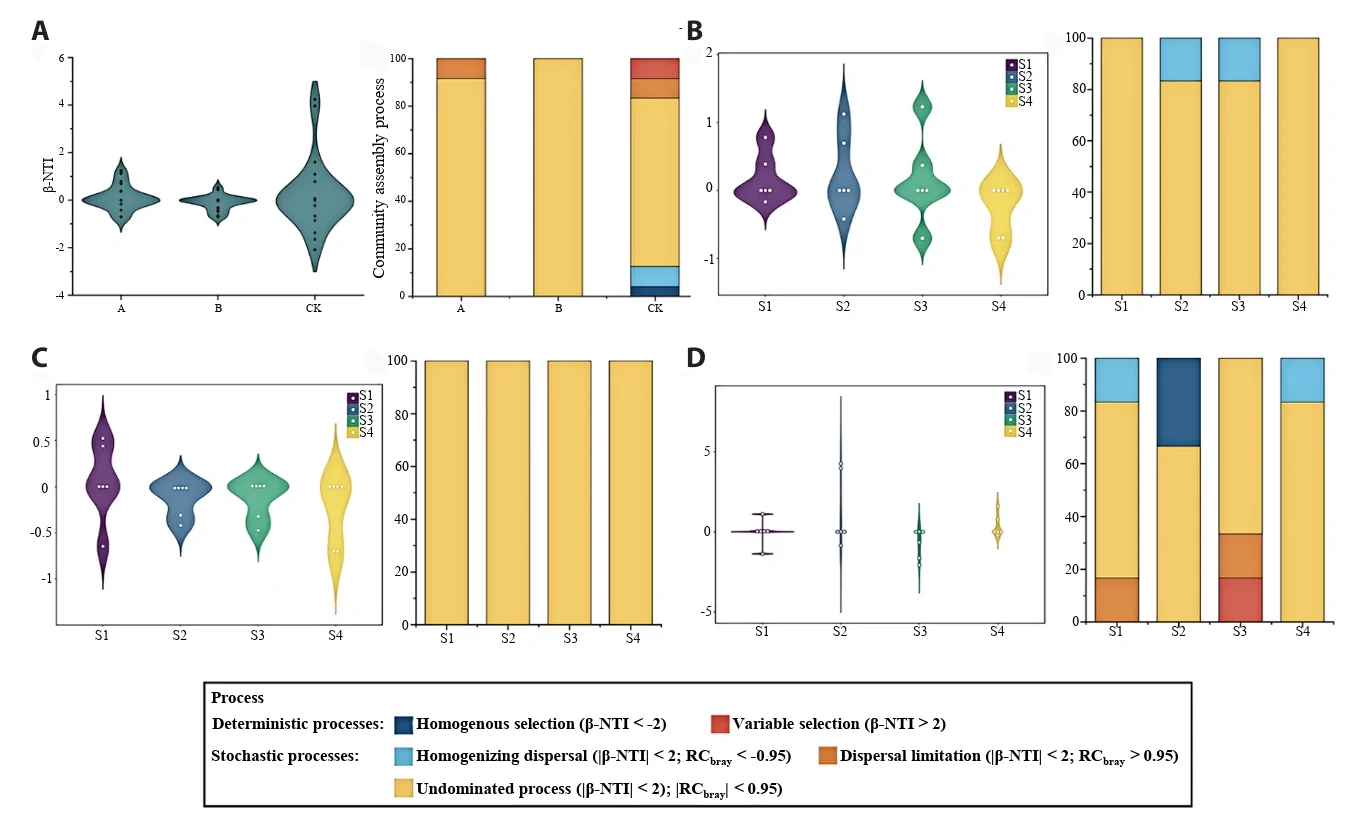
Assembling process of nitrogen-fixing microbial community structure under different periods and different treatments. (A) all treatment; (B) treatment A; (C) treatment B; (D) treatment CK.

The β-NTI and RCbray of the null model were calculated to evaluate the relative contributions of deterministic and stochastic processes to nitrogen-fixing microbial community assembly (Fig. 7). In all treatments, the β-NTI values of OTU-based communities predominantly fell within the range of -2 to 2, indicating that the assembly of nitrogen-fixing microorganisms was primarily governed by stochastic processes (Fig. 7A). Based on the RCbray value, it is found that undominated process plays a dominant role in the assembly process of nitrogen-fixing microorganisms (Fig. 7A), especially under the B treatment, the contribution of undominated process accounts for 100% (Fig. 7C). Deterministic processes occurred only under CK treatment, accounting for 33.33% at tiller stage and 16.67% at heading stage (Fig. 7D).

## Discussion

### Effects of AMD pollution on soil environmental factors

In this study, compared to control soils (CK), AMD-contaminated soils (Treatments A and B) exhibited significantly reduced pH (Xu et al., 2022) and elevated sulfate alongside heavy metal content (excluding Mn) (Mokgehle and Tavengwa, 2021; Wang et al., 2021). During the tillering stage (S2), a decrease in soil pH was observed across all experimental treatments. This phenomenon may be attributed to the elevated soil temperatures prior to July, which accelerated root growth and respiration, thereby enhancing the release of organic acids into the soil (Hicks Pries et al., 2017). The environmental impact of AMD contamination varies depending on its chemical composition and pH levels (Kefeni et al., 2017). Higher OM content observed in AMD-affected soils (A and B) relative to CK may stem from OM's role in inhibiting hydroxide formation and precipitation under low pH conditions (Michael et al., 2015). TP followed the trend A > B > CK, likely attributable to AMD-induced soil acidification promoting the binding of inorganic salts with Fe/Al oxides and hydroxides, thereby accumulating less bioavailable phosphates (Choudhury et al., 2017). As a major nutrient influencing plant growth, soil nitrogen dynamics were assessed via TN, NO_3_^⁻^-N, and NH_4_^+^-N. Higher TN and NO_3_^⁻^-N levels in Treatments A and B compared to CK suggest that AMD irrigation enhances organic nitrogen mineralization and inorganic nitrogen release, consistent with the negative correlation between soil pH and nitrification rates (Li et al., 2023a), which may explain increased NO_3_^⁻^-N in polluted soils. Notably, NH_4_^+^-N levels in CK soil were significantly higher than in A and B only during the heading stage, with minimal differences at other growth phases. This temporal pattern likely reflects elevated soil temperatures prior to July accelerating mineralization rates (Verburg, 2005), causing NH_4_^+^-N to peak during the tillering stage.

AMD irrigation significantly increased the concentrations of copper (Cu), cadmium (Cd), lead (Pb), and zinc (Zn) in the soil while showing no notable enrichment effect on manganese (Mn). The uptake of heavy metals by rice plants also exerted a measurable influence on the variation of soilheavy metal content (Miguel et al., 2017; Wang et al., 2018). In contrast, when contaminated soil was irrigated with clean water, the levels of Cu, Cd, and Zn showed a slight reduction compared to AMD irrigation, whereas Mn and Pb concentrations remained largely unchanged. This discrepancy may be attributed to the higher migration capacity of Cu, Cd, and Zn relative to Mn and Pb in the soil environment.

### Response characteristics of nitrogen-fixing microbial communities under different environmental factors

Our findings demonstrate that AMD pollution significantly affects nitrogen-fixing microorganisms in rice root zone soils, with clear differentiation between contamination levels and strong influence from both rice phenology and soil properties. This study is among the first to validate that AMD alters diazotrophic assembly processes in rice soils, with stochastic processes dominating even under attempted remediation (clean water irrigation).

The significantly higher α-diversity (Chao1 and Shannon indices) in CK soil compared to AMD-affected soils (A and B) underscores the inhibitory effect of AMD contamination on nitrogen-fixing communities (Dong et al., 2024). The lack of significant diversity difference between soils A and B suggests that while clean water irrigation (treatment B) may help restore certain community functions, it does not immediately recover microbial diversity to pre-contamination levels (Soleimani et al., 2023). This aligns with previous studies indicating that heavy metal pollution and low pH can cause long-term dysbiosis in soil microbiomes (Ma et al., 2024), even after external stress is partially mitigated. *Proteobacteria* dominated all treatments but was less abundant in contaminated soils (≥ 75% in CK vs. ~55–58% in A/B), indicating a shift toward acid- and metal-tolerant taxa under AMD stress (Chen et al., 2024). *Anaeromyxobacter* was also dominant across soils but declined over time in contaminated samples, unlike in controls. These patterns suggest AMD stress reduces key functional microorganisms (Dong et al., 2023).

Results from the LEfSe analysis revealed significant differences in nitrogen-fixing microorganisms among the different treatments. Taxa enriched in Soil A demonstrated acid-tolerant and metal-resistant properties, with *Sideroxydans* known for iron oxidation (Cooper et al., 2023) and *Pseudodesulfovibrio* involved in sulfate reduction (Bidzhieva et al., 2024). In Soil B, which underwent restoration treatment, the enrichment of *Methylococcus* and *Ideonella* may represent an early-stage recovery community. Specifically, *Methylococcus* has the capability to utilize methane (Esembaeva et al., 2024), and some strains of *Ideonella* can degrade pollutants (Michealsamy and Jayapalan, 2025), suggesting the emergence of new ecological niches following the reduction of contamination. In contrast, CK soil showed enrichment of genera such as *Bradyrhizobium* and *Nitrospirillum*, which are typically symbiotic or free-living nitrogen-fixing bacteria (Galanova et al., 2025), indicating that in healthy soil, microorganisms associated with nitrogen fixation can thrive and proliferate more effectively.

The variance partitioning analysis (VPA) and Mantel test results collectively highlight the predominant role of soil chemical properties over heavy metals in shaping the nitrogen-fixing microbial community structure and function (Wu et al., 2022; Xiang et al., 2024). The strong positive correlation between nitrogen-fixing microbes (at OTU level) and key soil properties—including TN, NO₃⁻-N, and pH—across all growth stages indicates that nutrient availability and soil acidity exert consistent selective pressure on community composition (Fan et al., 2024). The significant positive correlation between nitrogen-fixing microorganisms and heavy metals may be attributed to the dominance of metal-tolerant diazotrophs in AMD-contaminated soils. It is consistent with previous reports that low concentrations of heavy metals may act as trace nutrients stimulating the activity of diazotrophs (Shi and Ma, 2017).

Correlation heatmap analysis revealed that the majority of genera showed negative correlations with environmental factors. *Geobacter* is a metal-reducing bacterium known for its role in the reduction of Fe(III) and Mn(IV), which aligns with its observed positive correlation with Mn ions (Jiang et al., 2020). However, its significant negative correlations with most other environmental factors may result from ionic competition among metal elements. Both *Desulfovibrio* and *Pseudodesulfovibrio* are associated with sulfur reduction (Goñi-Urriza et al., 2020). *Dechloromonas*, on the other hand, is capable of perchlorate degradation (Shrout et al., 2004). They are all characterized by anaerobic metabolism and may exhibit enhanced performance under adverse conditions.

### Correlation and community assembly mechanisms among nitrogen-fixing microbial communities

The co-occurrence network facilitates visual analysis of inter-microbial relationships, where a greater number of connections indicates stronger microbial interactions (Munyai et al., 2024). Positive correlations among microorganisms may suggest nitrogen-fixing bacterial community co-presence or shared niche or similarity, while negative correlations could arise from competitive interactions or niche differentiation (Liu et al., 2020). The prevalence of positive correlations in the contaminated soil suggests that the majority of nitrogen-fixing microorganisms within it may exhibit tolerance to extreme environmental conditions characterized by high acidity and elevated metal concentrations (Chun et al., 2021). Results showed a general upward trend in the average connectivity of nitrogen-fixing microorganisms across all treatments as rice growth progressed. This likely reflects increasing structural complexity and diversity of nitrogen-fixing microbial communities influenced by plant development and dynamic soil conditions (Zhang et al., 2021). Notably, nitrogen-fixing bacterial genera exhibited tighter interactions under the control (CK) condition, potentially attributed to a more stable (Wendlandt et al., 2022) and favorable soil environment (Hyun et al., 2021). In contrast, AMD treatments reduced the average connectivity of inter-genus interactions, particularly in treatment A, likely due to environmental stress from contamination diminishing microbial richness and diversity, thereby weakening cooperative networks (Bao et al., 2020). The proportion of positive interactions among species decreased during the late rice growth stages. the plant transitions from vegetative to reproductive growth. The decrease in positive correlations during late growth stages probably indicates resource limitation and niche differentiation, as observed in other rhizosphere systems (Wang et al., 2023).

The assembly of ecological communities involves both stochastic processes and deterministic processes. Research (Wang et al., 2024b) demonstrated that soil bacterial community assembly is governed by niche-based deterministic and stochastic processes, with their relative influences shifting seasonally alongside vegetation dynamics. βNTI analysis revealed that stochastic processes predominate across all treatment conditions, indicating their greater importance compared to deterministic processes in the assembly of nitrogen-fixing microbial communities (Yang et al., 2025). In contrast to treatments A and B, only the control (CK) exhibited deterministic processes in soil bacterial community assembly, characterized by synergistic interactions of multiple mechanisms. This suggests that nitrogen-fixing microbial communities under control conditions are influenced by a broader range of ecological processes and demonstrate greater stability.

Conclusively, AMD pollution reduces diazotrophic diversity and destabilizes microbial networks, with stochastic processes driving the assembly of the microbial community. While clean water irrigation mitigates some impacts, it does not fully restore stability or diversity. Efficient remediation strategies should focus on reducing heavy metals, neutralizing acidity, and supporting functional recovery of nitrogen-fixing microorganisms. These results provide ecological understanding for managing AMD-affected agricultural soils and contribute to a broader knowledge of microbial resilience under extreme stress conditions.

## Supplementary Information

Table S1. Physicochemical properties and heavy metals of rice root zone soil under different treatments at different periods. Values are presented as Mean ± standard deviation. Different Letters indicate significant differences between samples at *p* < 0.05 using ANOVA test.

Table S2. Classification criterions for single factor index

Table S3. The degree of soil heavy metal pollution was analyzed by Single pollution index method

Table S4. The abundance of *nifH* gene in soil root zone in each sample (copies/g, Mean ± standard error). “p” represents the significant difference between different treatments in the same period.

Table S5. Correlation network index of nitrogen-fixing microorganisms in rice root zone in different samples

Fig. S1. Correlation network of dominant nitrogen-fixing microorganisms in rice root zone soil under A treatment. A connection represents a strong (|r| = 1) and significant (*p* < 0.05) correlation between genera.

Fig. S2. Correlation network of dominant nitrogen-fixing microorganisms in rice root zone soil under B treatment. A connection represents a strong (|r| = 1) and significant (*p* < 0.05) correlation between genera.

Fig. S3. Correlation network of dominant nitrogen-fixing microorganisms in rice root zone soil under CK treatment. A connection represents a strong (|r| = 1) and significant (*p* < 0.05) correlation between genera.
