# Supplementary material for: The impact of acid mine drainage on nitrogen-fixing microorganisms in rice root zone soil
Source: J Microbiol. 2026 Jan 31;64(1):e2505004. doi: 10.71150/jm.2505004 (PMC13577052; doi:10.71150/jm.2505004)
Supplement: Supplementary file 2 [file jm-2505004-Supplementary-Fig-S2.pdf]

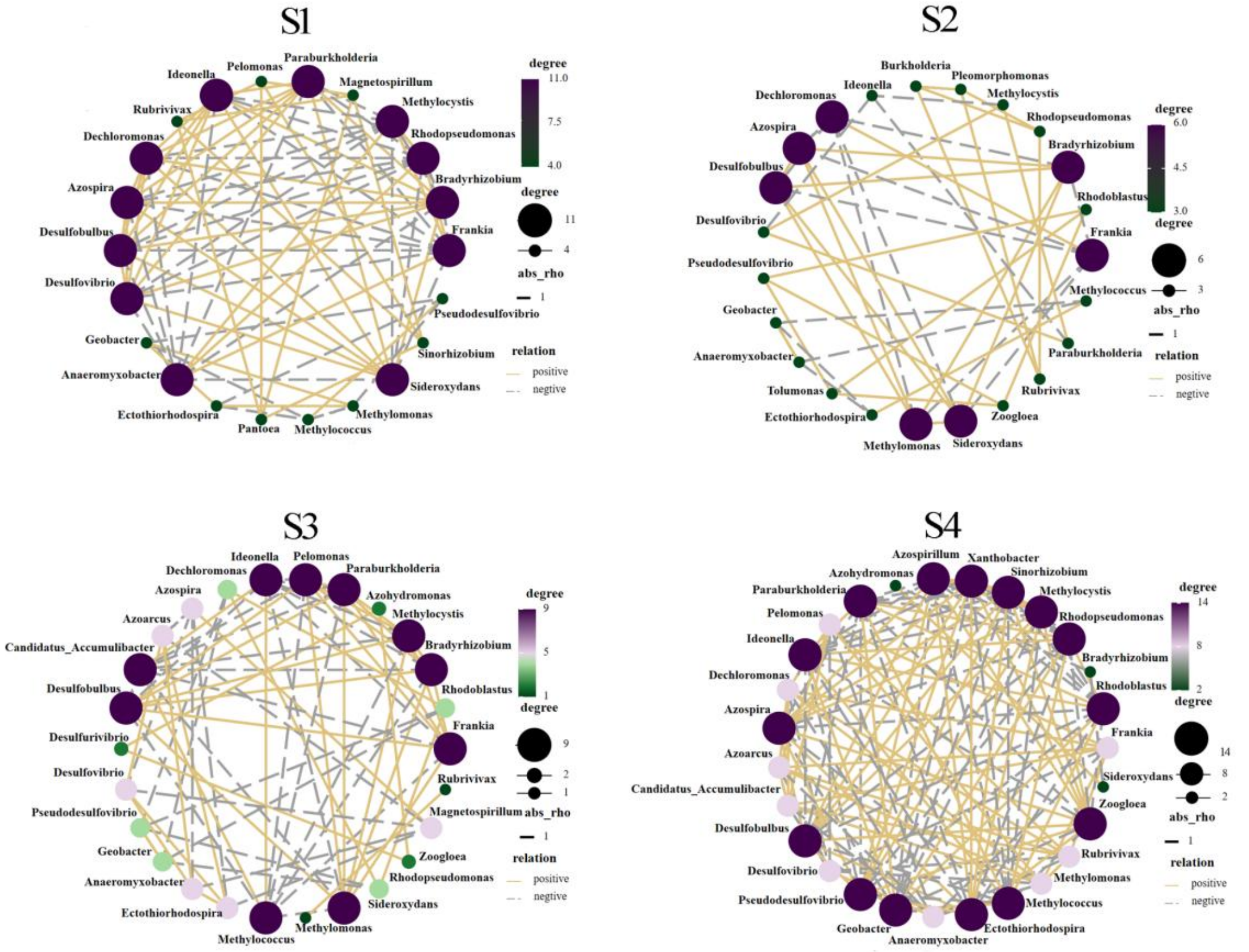


**Fig. S2.** Correlation network of dominant nitrogen-fixing microorganisms in rice root zone soil under B treatment. A connection represents a strong ($|r| = 1$) and significant ($p < 0.05$) correlation between genera.
