# Supplementary material for: The impact of acid mine drainage on nitrogen-fixing microorganisms in rice root zone soil
Source: J Microbiol. 2026 Jan 31;64(1):e2505004. doi: 10.71150/jm.2505004 (PMC13577052; doi:10.71150/jm.2505004)
Supplement: Supplementary file 3 [file jm-2505004-Supplementary-Fig-S3.pdf]

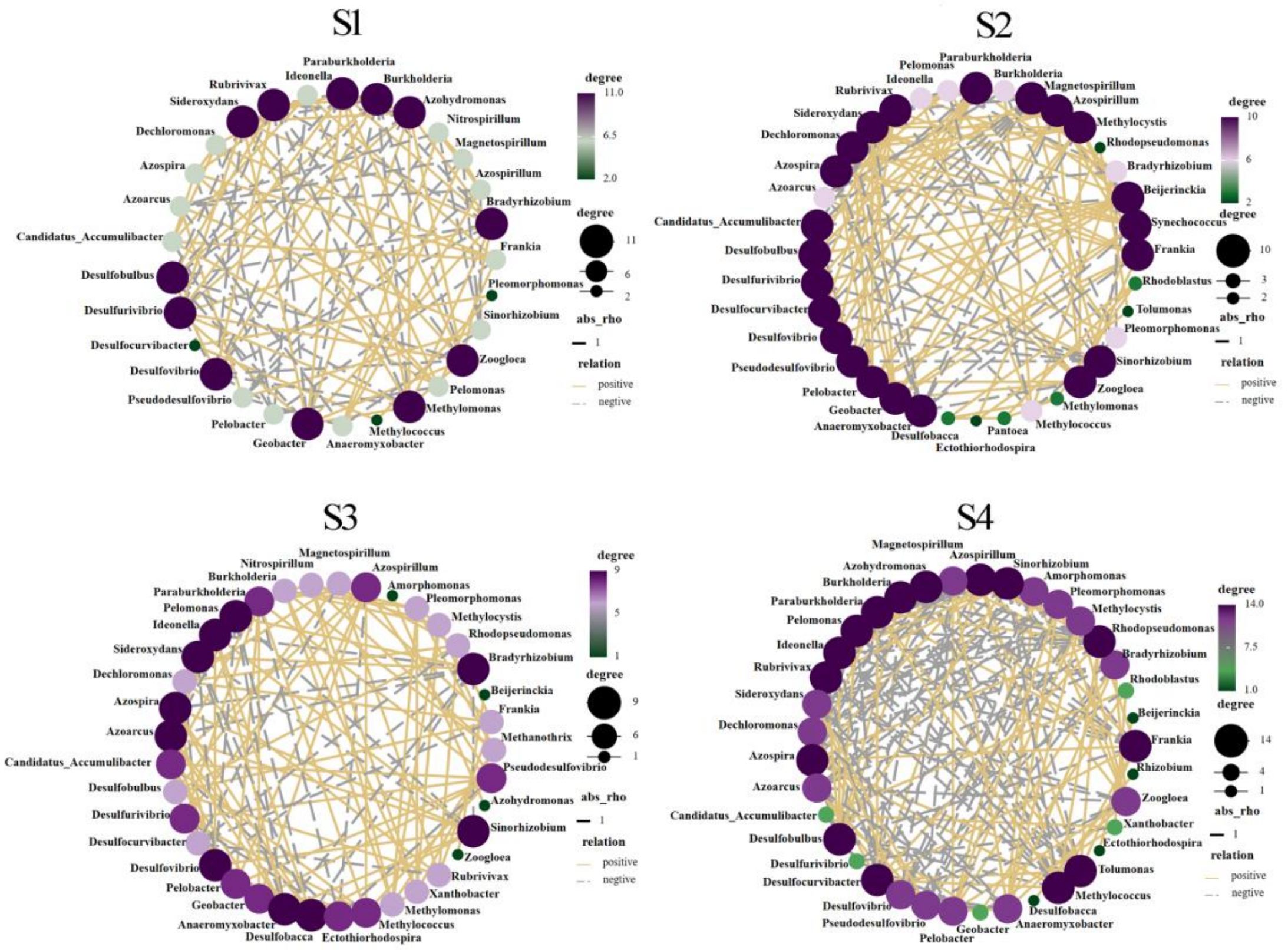

Fig. S3. Correlation network of dominant nitrogen-fixing microorganisms in rice root zone soil under CK treatment. A connection represents a strong (|r| = 1) and significant ($p < 0.05$) correlation between genera.
