# Supplementary material for: The impact of acid mine drainage on nitrogen-fixing microorganisms in rice root zone soil
Source: J Microbiol. 2026 Jan 31;64(1):e2505004. doi: 10.71150/jm.2505004 (PMC13577052; doi:10.71150/jm.2505004)
Supplement: Supplementary file 4 [file jm-2505004-Supplementary-Table-S1.pdf]

Table S1. Physicochemical properties and heavy metals of rice root zone soil under different treatments at different periods. Values are presented as Mean ± standard deviation. Different Letters indicate significant differences between samples at $p < 0.05$ using ANOVA test.

Treatment	Period	TN (g/kg)	NH_4^+-N (mg/kg)	TP (g/kg)	Cu (mg/kg)	Zn (mg/kg)	Mn (mg/kg)	Pb (mg/kg)	Cd (mg/kg)
A	S1	1.85 ± 0.13a	19.07 ± 1.99de	0.98 ± 0.03a	149.67 ± 4.56a	274.25 ± 0.68ab	171.79 ± 3.09b	243.65 ± 7.49bc	1.05 ± 0.02ab
	S2	1.85 ± 0.00a	27.64 ± 0.22b	0.95 ± 0.06ab	146.84 ± 5.36ab	277.35 ± 3.78a	174.61 ± 5.52b	208.03 ± 10.79d	1.16 ± 0.04a
	S3	1.62 ± 0.02bcd	16.4 ± 0.94e	0.82 ± 0.06cd	135.64 ± 2.07cd	277.47 ± 3.87a	177.17 ± 9.17b	260.96 ± 2.52ab	1.17 ± 0.01a
	S4	1.60 ± 0.04bcd	8.64 ± 0.15f	0.82 ± 0.06cd	154.69 ± 2.53a	276.87 ± 3.29a	157.33 ± 7.75b	267.53 ± 1.71a	0.99 ± 0.06b
B	S1	1.71 ± 0.11ab	21.16 ± 0.74de	0.87 ± 0.06bc	140.82 ± 0.69bc	260.84 ± 1.54d	172.92 ± 5.01b	237.27 ± 2.87c	0.93 ± 0.01b
	S2	1.69 ± 0.06abc	26.37 ± 1.96bc	0.82 ± 0.09cd	131.81 ± 0.84d	271.65 ± 3.05abc	166.84 ± 3.27b	211.77 ± 14.01d	0.91 ± 0.02b
	S3	1.54 ± 0.03cd	10.16 ± 0.35f	0.69 ± 0.05e	115.03 ± 3.20e	263.51 ± 1.59cd	165.06 ± 6.28b	250.37 ± 5.00abc	0.92 ± 0.04b
	S4	1.53 ± 0.08cd	7.54 ± 0.97f	0.74 ± 0.03de	140.82 ± 3.99bc	264.35 ± 5.52bcd	153.84 ± 2.85b	269.10 ± 0.89a	0.89 ± 0.06b
CK	S1	1.27 ± 0.03e	22.24 ± 3.69cd	0.51 ± 0.05f	28.08 ± 1.09f	96.47 ± 1.14e	363.17 ± 11.07a	35.69 ± 1.92e	0.65 ± 0.08c
	S2	1.38 ± 0.03d	48.92 ± 2.49a	0.55 ± 0.04f	27.75 ± 0.54f	97.29 ± 1.10e	378.51 ± 15.87a	30.71 ± 1.53e	0.68 ± 0.01c
	S3	1.18 ± 0.04e	10.77 ± 0.27f	0.58 ± 0.06f	26.35 ± 1.03f	99.19 ± 2.64e	367.14 ± 5.72a	33.72 ± 1.30e	0.66 ± 0.04c
	S4	1.14 ± 0.01e	8.54 ± 0.59f	0.59 ± 0.05f	27.50 ± 0.32f	96.98 ± 5.64e	360.20 ± 13.57a	32.05 ± 2.02e	0.66 ± 0.12c
