# Supplementary material for: The impact of acid mine drainage on nitrogen-fixing microorganisms in rice root zone soil
Source: J Microbiol. 2026 Jan 31;64(1):e2505004. doi: 10.71150/jm.2505004 (PMC13577052; doi:10.71150/jm.2505004)
Supplement: Supplementary file 5 [file jm-2505004-Supplementary-Table-S2.pdf]

Table S2. Classification criterions for single factor index

P_i	Contamination grade
$P_i \leq 1$	Non-pollution
$1 < P_i \leq 2$	Slight pollution
$2 < P_i \leq 3$	Light pollution
$3 < P_i \leq 5$	Moderate pollution
$P_i > 5$	Heavy pollution
