# Supplementary material for: The impact of acid mine drainage on nitrogen-fixing microorganisms in rice root zone soil
Source: J Microbiol. 2026 Jan 31;64(1):e2505004. doi: 10.71150/jm.2505004 (PMC13577052; doi:10.71150/jm.2505004)
Supplement: Supplementary file 6 [file jm-2505004-Supplementary-Table-S3.pdf]

Table S3. The degree of soil heavy metal pollution was analyzed by Single pollution index method

Treatment	Sample	P_iCu	P_iZn	P_iMn	P_iPb	P_iCd
A	S1	1.89	1.97	0.32	3.64	1.48
	S2	1.86	2.00	0.33	3.10	1.63
	S3	1.72	2.00	0.33	3.89	1.64
	S4	1.96	1.99	0.30	3.99	1.39
	Contamination grade	Slight pollution	Slight pollution	Non-pollution	Moderate pollution	Slight pollution
B	S1	1.78	1.88	0.33	3.54	1.31
	S2	1.67	1.95	0.31	3.16	1.29
	S3	1.46	1.90	0.31	3.74	1.30
	S4	1.78	1.90	0.29	4.02	1.25
	Contamination grade	Slight pollution	Slight pollution	Non-pollution	Moderate pollution	Slight pollution
CK	S1	0.36	0.69	0.69	0.53	0.92
	S2	0.35	0.70	0.71	0.46	0.96
	S3	0.33	0.71	0.69	0.50	0.93
	S4	0.35	0.70	0.68	0.48	0.93
	Contamination grade	Non-pollution	Non-pollution	Non-pollution	Non-pollution	Non-pollution
	Background value	79 mg/kg	139 mg/kg	530 mg/kg	67 mg/kg	0.71 mg/kg
