# Supplementary material for: The impact of acid mine drainage on nitrogen-fixing microorganisms in rice root zone soil
Source: J Microbiol. 2026 Jan 31;64(1):e2505004. doi: 10.71150/jm.2505004 (PMC13577052; doi:10.71150/jm.2505004)
Supplement: Supplementary file 7 [file jm-2505004-Supplementary-Table-S4.pdf]

Table S4. The abundance of *nifH* gene in soil root zone in each sample (copies/g, Mean ± standard error). "p" represents the significant difference between different treatments in the same period.

$nifH \times 10^5$				
Periods	A	B	CK	*p*
S1	5.11 ± 0.32a	1.53 ± 0.31b	1.07 ± 0.11b	< **0.001**
S2	3.27 ± 0.22a	2.73 ± 0.33a	0.58 ± 0.04b	< **0.001**
S3	6.70 ± 0.57a	5.08 ± 0.72ab	3.79 ± 0.49b	0.008
S4	2.99 ± 0.63b	7.95 ± 0.78a	2.02 ± 0.37b	< **0.001**
