# Supplementary material for: The impact of acid mine drainage on nitrogen-fixing microorganisms in rice root zone soil
Source: J Microbiol. 2026 Jan 31;64(1):e2505004. doi: 10.71150/jm.2505004 (PMC13577052; doi:10.71150/jm.2505004)
Supplement: Supplementary file 8 [file jm-2505004-Supplementary-Table-S5.pdf]

Table S5. Correlation network index of nitrogen-fixing microorganisms in rice root zone in different samples

Periods	Treatment	Number of nodes	Linking number	Average connectivity	Positive correlated connections	Negative correlated connections
S1	A	22	53	4.818	35	18
	B	22	86	7.818	48	38
	CK	29	111	7.655	61	50
S2	A	20	47	4.700	29	18
	B	23	45	3.913	28	17
	CK	36	140	7.778	94	46
S3	A	20	54	5.400	29	25
	B	26	74	5.692	39	35
	CK	37	125	6.757	90	35
S4	A	26	82	6.308	47	35
	B	27	144	10.667	74	70
	CK	37	195	10.541	95	100
